# Effects and Mechanisms of Low-Intensity Pulsed Ultrasound for Chronic Prostatitis and Chronic Pelvic Pain Syndrome

**DOI:** 10.3390/ijms17071057

**Published:** 2016-07-01

**Authors:** Guiting Lin, Amanda B. Reed-Maldonado, Maofan Lin, Zhongcheng Xin, Tom F. Lue

**Affiliations:** 1 Knuppe Molecular Urology Laboratory, Department of Urology, School of Medicine, University of California, San Francisco, CA 94143, USA; glin@urology.ucsf.edu (G.L.); amanda.reed-maldonado@ucsf.edu (A.B.R.-M.); 2 Department of Urology, Peking University First Hospital and the Institute of Urology, Peking University, Beijing 100009, China;maofanlin8473@163.com (M.L.); xinzc@bjmu.edu.cn (Z.X.)

**Keywords:** chronic prostatitis, chronic pelvic pain syndrome, low-intensity pulsed ultrasound, mechanism, therapeutic effect

## Abstract

Chronic Prostatitis/Chronic Pelvic Pain Syndrome (CP/CPPS) is one of the most common urologic diseases, and no curative treatments have been identified. Low-intensity pulsed ultrasound (LIPUS) has been successfully used in promoting tissue healing, inhibiting inflammation and pain, differentiating stem cells, and stimulating nerve regeneration/muscle regeneration, as well as enhancing angiogenesis. Very recently, LIPUS has been proven an effective approach for CP/CPPS. This review summarizes the possible mechanisms responsible for the therapeutic effect of LIPUS for CP/CPPS. To search publications relevant to the topics of this review, the search engine for life sciences of Entrez was used. We reviewed the available evidence from 1954 through 2015 concerning LIPUS for CP/CPPS. According to the literature, both transrectal and transperineal approaches of LIPUS are effective for CP/CPPS.

## 1. Introduction

Chronic Prostatitis (CP), one of the common urologic diseases, is frequently diagnosed in the field of andrology [1]. The majority of patients experience chronic pelvic pain without any evidence of infection; this is defined as Chronic Prostatitis/Chronic Pelvic Pain Syndrome (CP/CPPS) (category III of CP). However, the etiologies and mechanisms related to the pathogenesis of CP/CPPS are currently far from well understood, and no effective treatments have been successfully identified. Therefore, a novel and effective therapeutic approach is needed. Very recently, low-intensity pulsed ultrasound (LIPUS) has been successfully used in promoting tissue healing [2,3], inhibiting inflammation and pain [4], differentiating stem cells [5] and nerve /muscle regeneration [6], as well as enhancing cardiac angiogenesis [7] in other non-andrology fields. Since the first clinical application of ultrasound for CP conducted by Karpukhin et al. in 1977 [8], LIPUS has been proven an effective therapeutic approach for CP/CPPS in a randomized, double-blind, multi-center clinical trial [9]. Meanwhile, well-conducted experiments demonstrated several biological effects from LIPUS, such as regulation of cyclooxygenase-2 (COX-2) and monocyte chemoattractant protein 1 (MCP-1), which are responsible for this therapeutic effect and provided fundamental support for the application of LIPUS on CP/CPPS [10].

## 2. Chronic Prostatitis/Chronic Pelvic Pain Syndrome (CP/CPPS)

According to the National Institutes of Health (NIH) consensus classification of prostatitis syndromes, there are four clinical categories of prostatitis: (I) acute bacterial prostatitis; (II) chronic bacterial prostatitis; (III) chronic prostatitis/chronic pelvic pain syndrome (CP/CPPS); and (IV) asymptomatic inflammatory prostatitis. It is worth noting that the majority of CP patients are type III (about 90%–95%), without evidence of infection but with chronic pelvic pain. Clinically, this type of prostatitis is defined as chronic pelvic pain symptoms that last for at least three to six months without causes related to urinary tract infection or other causes, such as malignancy disease. It may be accompanied by urinary symptoms or sexual dysfunction, but organic or morphological local change does not explain the chronic pain in these CP patients. In general, CP/CPPS is associated with many symptoms, such as pelvic pain, irritative and obstructive voiding symptoms, and sexual dysfunction. Worldwide, 2% to 14% of men may suffer the symptoms of CP/CPPS [1].

Several biological mechanisms have been reported for CP/CPPS, such as infection, immunological abnormality, neurological dysfunction, psychosocial problems, and endocrine disorders. However, the etiology and pathogenesis of CP/CPPS remain poorly understood [11]. Notably, organic or morphological pathology does not explain the chronic pelvic pain, which is the dominant complaint of CP/CPPS patients. Recently, it has been reported that central sensitization (CS) is also related to CP/CPPS, and this may be the cause of chronic pain. CS is defined as an augmentation of responsiveness of central cortical neurons to input from unimodal and polymodal receptors. Korkmaz conducted a clinical experiment in 17 male patients diagnosed with CP/CPPS and 17 healthy male controls and demonstrated that CS may be a possible etiological factor responsible for the pain sensation in those patients. In the study, an electrical stimulus was applied with penile ring electrodes for somatosensory evoked potentials recording, and latency of N50 was defined as the second negative (upward) deflection of the W-shaped averaged cortical waveform. Results indicated that N50 latencies were significantly shortened in the CP/CPPS patient group compared to the healthy controls (*p* < 0.001). Therefore, normalization of transmission might be an important step in treatment of pain in patients with CP/CPPS [12]. Updated research confirmed that LIPUS promoted nerve regeneration in vivo [6].

Importantly, in addition to the dominant chronic pelvic pain symptoms, CP/CPPS also negatively impacts the quality of life of men in many aspects, such as erectile function. In the clinic, erectile dysfunction (ED) is another major concern for CP/CPPS patients. ED is defined as the persistent inability to attain and maintain a penile erection that is sufficient for satisfactory sexual performance [13]. Though the underlying mechanisms are unclear, recent research suggested a link between CP/CPPS and ED, which is related to the arterial stiffness or endothelial dysfunction, as well as psychological factors including stress, depression, and anxiety.

It is worth noting that CP/CPPS is a complex clinical condition with wide criteria and currently lacks well-validated diagnostic biomarkers [14]. Very recently, several biomarkers, including Interleukin 8 (IL8), MCP-1, and macrophage inflammatory protein-1α (MIP-1α), have been proven to be strongly correlated with CP/CPPS. IL-8 is related to several diseases, such as abdominal aortic aneurysms, rheumatoid arthritis, inflammatory bowel disease, gastritis, and lung disease [15,16]. Recently, it has been reported that the IL-8 value is strongly correlated with CP/CPPS (*p* < 0.001); the patients with the worst symptoms have higher levels of IL-8 reported [15]. At the same time, MCP-1 and MIP-1α recruit monocytes and macrophages, which may also be responsible for CPPS as this subtype of CP patients had statistically higher levels of MCP-1 and MIP-1α than the control group or patients with benign prostatic hyperplasia.

Although several etiologies and mechanisms have been proposed for the pathogenesis of CP/CPPS, the real cause of this disease remains unknown, and effective treatments have not yet been identified. Therefore, available therapeutic options for CP/CPPS are far from satisfactory for physicians or for patients. A novel, effective therapeutic approach is desperately needed [1].

## 3. Therapeutic Ultrasound and Low-Intensity Pulsed Ultrasound (LIPUS)

The application of ultrasound in medicine has developed in two directions: the diagnostic imaging modality and therapeutic approach in which energy is deposited into target organs and tissues to induce biological effects that can be appreciated clinically. Recently, developments in the science of ultrasound have improved the latter application and made ultrasound a possible therapy for various diseases [17,18,19,20].

An ultrasound wave is a high-frequency wave that is generally 1–12 MHz. Ultrasound waves transmit through body tissues where they induce molecular vibration and collisions leading to biological responses and events at both cellular and molecular levels. According to the level of ultrasonic energy, therapeutic ultrasound can be classified into two categories: high-intensity ultrasound with peak intensities of 5000–15,000 W/cm^2^ and low-intensity ultrasound with intensities of 0.5–3000 mW/cm^2^.

Recently, LIPUS at low intensity (<0.1 W/cm^2^) and a constant frequency (1–1.5 MHz), which reduces any significant thermal effect, has been widely utilized to promote tissue healing, inhibit inflammation and pain, treat CP/CPPS, activate stem cell and nerve and muscle regeneration, and enhance cardiac angiogenesis. However, the potential mechanisms producing the above biological effects are still unclear and are under further investigation.

## 4. Application of LIPUS for CP/CPPS

In 2013, Li et al. [9] evaluated the clinical efficacy and safety of transperineal ultrasonic therapy with a Therapeutic Ultrasonic Device (GR-QLX, Beijing Guorui Huihuang Medical, Ltd., Beijing, China) for CP by analyzing the National Institutes of Health Chronic Prostatitis Symptom Index (NIH-CPSI) scores along with routine prostate examination and evaluation of expressed prostatic fluid. They conducted a randomized, double-blind, multi-center trial on 96 CP patients. The patients were divided into groups A (trial) and B (control) of equal number; the former were treated with transperineal LIPUS and the latter with the same machine but no ultrasound waves. The treatment protocol was 10 min per treatment daily for two weeks. The therapeutic effect and safety were evaluated by comparing the NIH-CPSI scores and counts of white blood cells (WBC) and lecithin corpuscles (LC) in the prostate fluid between the two groups before and after treatment. Results demonstrated that the total effectiveness rate was 70.83% in group A and 25% in group B (*p* < 0.01). The scores on prostate pain, urinary symptoms, and quality of life, as well as the total NIH-CPSI score, were significantly improved in group A as compared with pre-treatment (*p* < 0.05), and so were the prostate pain score and total NIH-CPSI score in group B (*p* < 0.05). Statistically significant differences were observed between the two groups in the scores on prostate pain and urinary symptoms and total NIH-CPSI score after treatment (*p* < 0.05), but not in any of the NIH-CPSI scores before treatment (*p* > 0.05), nor were there any significant differences in the counts of WBCs and LC either between the two groups or within each group before and after treatment (*p* > 0.05).

Results demonstrated that transperineal ultrasonic therapy is highly effective for CP, especially in relieving prostate pain. With its advantages of safety, easy operation, and high acceptability, LIPUS was recommended for a wider clinical application. Transrectal LIPUS has also been previously reported to be effective in improving clinical symptom of CP/CPPS [8].

### 4.1. Physical Mechanisms of LIPUS for CP/CPPS

When LIPUS is applied for therapeutic usage, the ultrasonic energy is absorbed at different rates depending on the density of the tissue through which the ultrasound waves pass. The potential biological actions of ultrasound are produced through two major physical mechanisms: thermal effects and non-thermal effects, which include acoustic cavitation and mass transfer enhancement.

#### 4.1.1. Thermal Effect

During the application of LIPUS, the ultrasonic waves propagate though the body, and the ultrasonic energy is absorbed according to the density of the tissue through which the waves pass and the density of the target tissue. In general, the absorption of the ultrasound signal results in an increase in the temperature of the target tissue. Although this thermal effect from LIPUS is rather minor, some enzymes, such as MMP-1 and collagenase, are exquisitely sensitive to even extremely minor changes of temperature [21] and function will be affected. Thermal deactivation is one of the important mechanisms in the denaturation of enzymes induced by LIPUS.

#### 4.1.2. Cavitation

One of the dominant non-thermal effects in target tissues from the ultrasound energy of LIPUS is the cavitation phenomenon [22]. There are two different types of acoustic cavitations: stable cavitation and transient cavitation. The stable cavitation produce bubbles which present for a great number of acoustical cycles, and the radius of every bubble varies about an equilibrium value. On the other hand, the transient cavitation forms bubbles which oscillate in an unstable manner and expand to two or three times their resonant size before collapsing violently. Both types of cavitation are considered to be main mechanisms for the biological effects on target tissues, while the transient cavitation actions are responsible for the damage to intact cells.

#### 4.1.3. Mass Transfer Enhancement

It is well known that ultrasound increases the movement of the liquid medium, precipitating mass transfer and reaction rates, which also occurs in target tissue and cells treated with LIPUS. In general, this happens in three main areas, including the cell membrane, the cytosol, and the boundary layer. A micro-stream around an acoustic field is generated by the vibratory gas bubble and leads the reagents to the active site of the enzyme or to the cell. Meanwhile, the biological products are released into the medium, where the biological effect occurs.

### 4.2. Biological and Molecular Mechanisms of LIPUS for CP/CPPS

**Figure 1:**
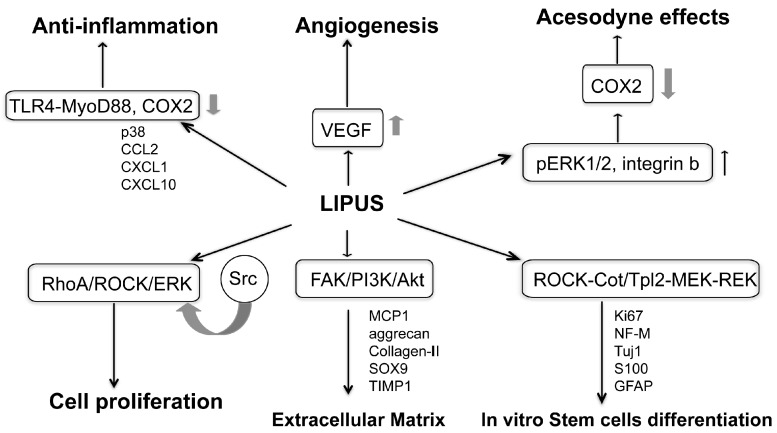
Biological and molecular mechanisms of Low-intensity pulsed ultrasound (LIPUS) for the therapy of chronic prostatitis/chronic pelvic pain syndrome (CP/CPPS). Upward arrow: increase; Downward arrow: decrease.

Extensive research demonstrates that LIPUS results in minor heat and other biological signaling in the target tissues. Therefore, LIPUS may re-establish or normalize the effective metabolic temperatures in prostatic tissue-healing regions [23]. Though this effect is subtle, its biological effect may be profound. Furthermore, at interfaces of distinct densities, the energy of LIPUS is reflected and results in complex gradients of acoustic pressure and biological response through the tissue. LIPUS stimulates many biological events that may be of treatment benefit in CP/CPPS, including gene expression, cell signaling transduction, enzymatic activity, cell proliferation and differentiation, cytokine secretion, angiogenesis, anti-inflammation and acesodyne effect, and stem cell differentiation (Figure 1).

#### 4.2.1. Cell Signaling Pathways Affected by LIPUS

##### Activation of Rho A/ROCK/ERK Pathway

In 2004, by applying LIPUS to primary human foreskin fibroblasts, Zhou et al. investigated the effects of LIPUS at an intensity of 0.03 W/cm^2^ [24]. They noted that LIPUS activated Rho-associated coiled—coil-containing protein kinase (ROCK)-dependent pathway. Furthermore, daily LIPUS exposure resulted a two-fold increase in extracellular signal related kinase (ERK) 1/2 activation, as well as triggered cell proliferation. Actually, the RhoA/ROCK is an upstream regulator of the LIPUS-induced ERK pathway. LIPUS also triggered Src, which further regulates the ERK cascade.

##### Activation of FAK/PI3K/Akt Pathway

It is well known that the extracellular matrix is very important in maintaining a normal morphology and function in both organs and tissues, especially of the prostate. In 2015, Zhang et al. [25] explored the effect of LIPUS on another cell type, the human nucleus pulposus cells, and confirmed that LIPUS significantly up-regulated expression of aggrecan, collagen-II, Sox9, and tissue inhibitor of metalloproteinase-1 compared to the control group, but it inhibited secretion of matrix metalloproteinase-3. The study further demonstrated that the up-regulation of aggrecan, collagen-II, and Sox9 was related to the activation of FAK/PI3K/Akt pathway caused by LIPUS. Inhibition of PI3K/Akt significantly suppressed the special biological effect activated by LIPUS [25].

This effect was also confirmed by Cheng’s group [26]. They demonstrated the effect of LIPUS on extracellular matrix (ECM) production via modulation of the integrin/focal adhesion kinase (FAK)/phosphatidylinositol 3-kinase (PI3K)/Akt pathway. These authors showed that LIPUS may affect the integrin-FAK-PI3K/Akt mechanochemical transduction and alter ECM production.

#### 4.2.2. Potential Genes Affected by LIPUS

With the high throughout microarray platform, the genes affected by LIPUS were extensively studied. There are 38 genes upregulated and 37 genes downregulated by 1.5-fold or more, which were identified in the cells after LIPUS treatment. It was reported that seventeen genes were validated by real-time quantitative PCR assay. In addition to the up-regulated genes, many down-regulated genes were also affected by LIPUS. Interestingly, this network contained the inhibitor of differentiation (Id) genes, including Id1, Id2 and Id3, which belong to the helix-loop-helix (HLH) transcription factors that can form heterodimers among the basic HLH transcription factors. These genes are likely to be involved in the acceleration of tissue healing induced by LIPUS.

Kobayashi [27] also applied cDNA microarray to assess the genes affected by LIPUS in vitro. Their cDNA array results confirmed that LIPUS significantly stimulated the expression of growth factors, including BMP2, FGF7, TGF β R1 EGFRF1, and vascular endothelial growth factor (VEGF), and their receptors. In 2008, Omi et al. [10] reported that LIPUS stimulates cell proliferation and proteoglycan production in rabbit intervertebral disc cells and promotes the secretion of MCP-1 from macrophages. However, they did not assess the dosage response. The possible application of LIPUS for biological repair could be achieved based upon those biological effects, though the mechanisms involved are not well understood. Therefore, in their experiment to evaluate the effect of LIPUS stimulation on cytokine production, in vitro culture studies of nucleus pulposus cells and macrophages were conducted. After culture and stimulation with LIPUS for seven days, the culture medium and the cells were analyzed by cytokine array, RT-PCR, and ELISA. Results demonstrated that LIPUS stimulation significantly up-regulated TIMP-1 in the nucleus pulposus and MCP-1 in macrophages compared to that in the control. The result was confirmed by RT-PCR and quantitative evaluation of proteins by ELISA.

### 4.3. LIPUS Promotes Angiogenesis through VEGF

Several reports claimed an angiogenesis effect from LIUPS, which may promote resolution of symptoms in CP/CPPS patients. To figure out the mechanism of this biological effect, Hanawa et al. [7] assessed expression of VEGF in human umbilical vein endothelial cell (HUVEC). They confirmed that LIPUS significantly up-regulated mRNA expression of VEGF in cultured human endothelial cells, and this effect depended on the ultrasound cycles and the number of ultrasound waves in each pulse. The peak occurred at 32 cycles. They also examined the in vivo effects of LIPUS in a porcine model of chronic myocardial ischemia with reduced left ventricular ejection fraction (LVEF). Interestingly, the results indicated that the capillary density in the ischemic region of heart was significantly improved in the LIPUS group compared with the control group.

### 4.4. Effect of LIPUS on in Vitro Differentiation of Stem Cells

In 2013, Lv et al. [5] explored the effect of LIPUS on induced pluripotent stem cell-derived neural crest stem cells (iPSCs-NCSCs) by checking the cell proliferation, cell viability, and differentiation of iPSCs-NCSCs. Results indicated that LIPUS at 500 mW/cm^2^ enhanced the viability and proliferation of iPSCs-NCSCs after two days of LIPUS and up-regulated the expression of GFAP, S100β, Tuj1, and NF-M in iPSCs-NCSCs after four days of LIPUS. At seven days post LIPUS, only GFAP, NF-M, and S100β were up-regulated. Their results demonstrated that the LIPUS could be an efficient and cost-effective method to enhance cell proliferation, cell viability, and neural differentiation in vitro, which may be of benefit for peripheral nerve tissue engineering. The same group confirmed this beneficial effect in vivo in 2015 [6]. They applied the iPSCs-NCSCs as a bridge in rat transected sciatic nerve and found treatment with 0.3 W/cm^2^ LIPUS for two weeks and 5 min per day significantly improved the sciatic functional index. Histological analysis also revealed new blood vessels and new neurofilaments, and higher expression level of β-III tubulin (Tuj1) was noted in the experimental group seeded with iPSCs-NCSCs and stimulated with LIPUS.

Further experiment demonstrated the effect of LIPUS on stem cells is related to ROCK-Cot/Tpl2-MEK-ERK pathway. In 2014, Kusuyama found LIPUS can influence the multilineage differentiation of mesenchymal stem and progenitor cell lines via ROCK-Cot/Tpl2-MEK-ERK signaling pathway [28]. In this study, LIPUS was applied to adipogenic progenitor cell and mesenchymal stem cell (MSC) lines to analyze how cell differentiation was affected. Impressively, the adipogenic differentiation of both cell types was suppressed by LIPUS and was represented by impaired lipid droplet appearance. In addition, expression of peroxisome proliferator-activated receptor γ2 (Pparg2) and fatty acid-binding protein 4 (Fabp4) were decreased in the LIPUS-treated group. On the contrary, LIPUS promoted the MSC line differentiation into osteogenic cells by inducing the expression of runt-related transcription factor 2 (Runx2) and Osteocalcin. It was also noted that LIPUS could induce the expression of phosphorylation of cancer Osaka thyroid oncogene/tumor progression locus 2 (Cot/Tpl2) kinase and enhance the phosphorylation process of mitogen-activated kinase kinase 1 (MEK1) and p44/p42 extracellular signal-regulated kinases (ERKs). This effect could be blocked by a Cot/Tpl2-specific inhibitor. Therefore, the LIPUS could suppress adipogenesis and promote osteogenesis of MSCs through the signaling pathway of Rho-associated kinase-Cot/Tpl2-MEK-ERK [28].

### 4.5. Anti-Inflammatory Effect from LIPUS through TLR4 and COX2

In 2014, with a LPS-induced inflammation model, Nakao et al. [4] explored the effect of LIPUS to determine its mechanism of action. In their experiment, LPS induced the expression of CXCL1, CCL2, and CXCL10, while LIPUS treatment significantly inhibited the expression of CXCL1 and CXCL10 induced by LPS. For the mechanism, LIPUS significantly decreased the phosphorylation of ERKs, p38 kinases, MEK1/2, MKK3/6, IKKs, TBK1, and Akt induced by LPS as well. Meanwhile, LIPUS inhibited the transcriptional activation of NF-κB responsive element and interferon-sensitive response element (ISRE). Additionally, in an experiment of transient transfection, LIPUS significantly inhibited the formation of TLR4-MyD88 complex. Therefore, the anti-inflammatory effect from LIPUS was through inhibiting TLR4 signal transduction in the target cells.

An in vivo experiment also confirmed the anti-inflammatory effects from LIPUS in MRL/lpr mice [29]. It has been reported that stimulation with pro-inflammatory cytokines significantly promoted cell proliferation, which was significantly decreased in the LIPUS exposure group. In this in vivo experiment, LIPUS treatment resulted in a significant reduction of histological damage in MRL/lpr mice compared to that in control. Impressively, the Cox-2-positive cells were markedly decreased in the animals treated with LIPUS. The study demonstrated that LIPUS treatment might be an effective therapy for inflammatory diseases, such as CP.

### 4.6. Acesodyne Effects from LIPUS for CP/CPPS

The dominant symptom of CP/CPPS is chronic pelvic pain. It is well known that inhibition of COX-2 can provide relief for inflammation and pain. In 2014, to determine the effect of LIPUS on the expression of COX-2 and related mechanisms, Iwabuchi et al. [30] used the articular chondrocytes primarily derived from porcine mandibular condyles after the treatment of interleukin-1β (IL-1β) and treated with LIPUS for 20 min. The conditioned medium was changed to a fresh one without IL-1β post-LIPUS, and the cells were cultured for 0 to 24 h. The expression of COX-2, p-integrin β1, and phosphorylated extracellular signal-related kinase (p-ERK½) were checked with real-time PCR and Western blot. The results indicated that LIPUS significantly decreased the COX-2 mRNA level induced by IL-1β. Furthermore, phosphorylation of integrin β and expression of p-ERK½ were also inhibited by LIPUS significantly. Therefore, COX-2 expression could be inhibited by LIPUS through a mechanism of the integrin β1 receptor followed by the phosphorylation of ERK½. This demonstrated that LIPUS is a potential candidate as a therapeutic approach for CP/CPPS.

## 5. Prospective

LIPUS is regarded as effective clinical procedure for the treatment of CP/CPPS for many reasons. LIPUS is a non-invasive treatment with anti-inflammatory, acesodyne effects through pathways including regulation of gene expression, cell signaling transduction, impacts on enzymatic activity, cell proliferation and differentiation, cytokine secretion, angiogenesis, and stem cell differentiation. Overall, LIPUS is safe for both the operator of the equipment and patients treated [31].

Both transrectal and transperineal approaches are effective. With more basic and clinical research, the mechanism of LIPUS for its biological effects will be further clarified and optimal clinical energy dosage and therapeutic protocols will be established. This will extensively enhance the clinical outcomes for patients with CP/CPPS treated with LIPUS.
